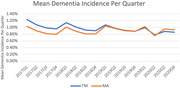# Changes to Medicare Advantage Risk Adjustment and Dementia Diagnosis in Older Adults

**DOI:** 10.1002/alz.089359

**Published:** 2025-01-09

**Authors:** Sidra Haye, Mireille Jacobson, Geoffrey Joyce, Julie M Zissimopoulos

**Affiliations:** ^1^ University of Southern California, Los Angeles, CA USA

## Abstract

**Background:**

Diagnosing dementia supports patients’ access to treatment options and social services. The addition of dementia to the risk adjustment model for Medicare Advantage (MA) plan payments, affecting claims beginning in 2019, provided an incentive for MA plans to improve the detection and diagnosis of dementia among their enrollees. The objective of this project is to examine the changes in annual incident dementia diagnoses rates associated with the inclusion of dementia diagnoses in risk adjustment models for Medicare Advantage.

**Methods:**

We conducted a retrospective study using 2017‐2020 data for community‐dwelling beneficiaries enrolled in TM and MA. We estimated difference‐in‐differences (DiD) models to examine changes in incident dementia diagnosis rates in MA relative to TM before and after 2019. The rate of incident dementia diagnosis in each year among beneficiaries without any dementia diagnosis in the year prior to the year of interest.

**Results:**

There were 84,997,374 beneficiaries. In regression models that adjusted for beneficiary characteristics and time trends, we find that in response to the payment change, the annual incident dementia diagnosis rates in MA increased by about 0.282 percentage points or 11.5% relative to TM. We further find that the increase in incident dementia diagnosis was concentrated among beneficiaries who were more likely to have undiagnosed dementia, specifically, beneficiaries who were Hispanic or Black, who were ages 85 and over, and who were dual‐eligible/low‐income subsidy. Sensitivity analyses excluding 2020 data and allowing beneficiaries to switch between TM and MA during our sample years finds similar results.

**Conclusion:**

Financial incentives to detect dementia resulted in more dementia diagnoses, particularly among persons at high risk of dementia and of undetected dementia but also raises concerns about diagnostic upcoding and the potential for over‐diagnosis.